# Genome-wide differential mRNA expression profiles in follicles of two breeds and at two stages of estrus cycle of gilts

**DOI:** 10.1038/s41598-017-04336-x

**Published:** 2017-07-11

**Authors:** Qingpo Chu, Bo Zhou, Feilong Xu, Ruonan Chen, Chunyan Shen, Tingting Liang, Yuan Li, Allan P. Schinckel

**Affiliations:** 1 Department of Animal Genetics, Breeding and Reproduction, College of Animal Science and Technology, Nanjing Agricultural University, Nanjing, 210095 P.R. China; 2 Department of Animal Sciences, Purdue University, West Lafayette, IN 47907-2054 USA

## Abstract

Estrus expression by gilts and sows is hereditable and important for heat detection. To better understand the molecular biological mechanisms of estrus expression in gilts, the mRNA expression profiles of follicular tissue from Large White gilts in diestrus (LD, n = 3) and estrus (LE, n = 3), and Chinese indigenous Mi gilts in diestrus (MD, n = 2) and estrus (ME, n = 3) were investigated using RNA sequencing. We detected 122,804-335,295 SNPs, 6,140-14,947 InDel and 12 types of AS events (39.57% TSS, 34.90% TTS) in 11 samples. A total of 2,838 differentially expressed genes (DEGs) were found in LD vs MD, LE vs ME, LE vs LD, or ME vs MD comparisons. Two DEGs (*ACP5* and *PIGS*) were observed in all comparisons. Two new genes (ENSSSCG00000028235 and ENSSSCG00000021903) were exclusively expressed in Mi and Large White gilts, respectively. Bioinformatics analyses indicate that these DEGs are involved in single-organism process, catalytic activity, cell adhesion and enriched in ECM-receptor interaction, olfactory transduction, ovarian steroidogenesis, steroid biosynthesis and CAMs signaling pathways. These results of RNA-Seq have provided important information for screening the key functional genes or molecular markers of estrus expression in gilts.

## Introduction

Clear visible estrus behaviors, such as standing reflex, reddening and swelling of the vulva, mucus discharge from the vulva, etc., contribute to the reproductive performance of sows^1^. However, the proportion of gilts and sows that do not express estrus behaviors is increasing in recent years^2–4^. Estrus expression of gilts or sows is associated with clearly visible physiological and behavioral changes^5^, which reach the maximum intensity before the onset of estrus^6^. Reproductive behavior culminates in the standing reflex, which indicates sexual receptivity^6, 7^. Previous studies showed that Chinese indigenous pigs which originated in Taihu Lake Basin, such as Meishan, Erhualian, and Mi pigs, had superior reproductive performance and estrus expression traits than European pigs^8–11^. The difference on estrus expression between pig breeds indicates that the clear visible expression of estrus is heritable^1^, and could be improved by selection^12, 13^. A positive correlation was observed between the serum estrogen concentration of gilts and the intensity of estrus behaviors^14–16^. The synthesis of ovarian estrogen is under the action of luteinizing hormone (LH) and follicle-stimulating hormone (FSH) in the theca cell and granulosa cells^17^. During the growth and development of follicles, LH stimulates the theca interna to secrete testosterone, and then under the stimulation of FSH, granulosa cells convert testosterone to estradiol, namely “double cell dual-sex hormone effect mode”^17^.

The plasma concentration of estrogen is affected by the dynamic equilibrium of estrogen synthesis and metabolism, which requires the activity of enzymes, such as cytochrome P450 19A1 (*CYP19A1*), estradiol 17-beta-dehydrogenase 12 (*HSD17B12*), sulfotransferase family 1 C member 3 (*SULT1C3*), etc^18, 19^. Previous studies found that differentially expressed genes (DEGs)), were related to follicular development and hormone metabolism^20–22^.

Chinese indigenous Mi pigs, as well as Meishan and Erhualian pigs, originated in the lake Taihu basin in Jiangsu province (east China)^23^. These Chinese pig breeds have similar biological characteristics^24^, such as greater litter size and clearer expression of estrus behaviors. Gilts of these Chinese pig breeds reach puberty at an earlier age^25^, express behavioral estrus longer, and have slightly shorter estrus cycles^8^ than those of Landrace and Large White. The duration of high estradiol concentrations was greater in Meishan than in Large White hybrid gilts during the follicular phase, the periovulatory period, and the early luteal phase^26^.

In the present study, we investigated the mRNA expression profiles of follicular tissue from Large White gilts at diestrus (LD) and estrus (LE), and Mi gilts at diestrus (MD) and estrus (ME) using RNA-seq. The goals of this study were to screen genes and new transcripts that are differentially expressed between two breeds or/and two stages of estrus cycle, and to find which biological processes and pathways have the greatest number of DEGs which could contribute to identifying molecular genetic markers associated with the expression of estrus in pigs.

## Results

### Sequencing data summary

A sample from a Mi gilt at diestrus was discarded because of degradation. We generated 11 RNA-seq libraries of follicle tissue from six Large White gilts and five Mi gilts. Utilizing paired-end Illumina sequencing technology, about 630 million raw reads were obtained, and 610 million high-quality reads were obtained after removing low quality reads. The number of clean reads was more than 54 million in each group (Table 1). The ratio of Q30 (with a base quality > 30 and error rate < 0.001) bases comprised more than 87% clean data. In four groups, 77.33% to 80.55% clean reads were mapped onto the *Sus scrofa* reference genome (sscrofa10.2), and 4.33% to 5.00% clean reads were multi map reads (Table 1). The genic distribution of clean reads was comprised of 66.95% exonic, 8.02% intron, and 25.03% intergenic regions (Supplementary Table S3). To ensure the reliability of the analysis results, the unique mapped reads were used for subsequent statistical analysis. Reads per kilobase millon mapped reads (RPKM), a normalized transcription level, can eliminate the influence of gene length and sequence difference on gene expression^27^. Therefore, here RPKM was used to compare the gene expression between different samples.

**Table 1:** Quality analyses of the digital gene expression profiling library of follicles tissue in Large white and Mi gilts at estrus and diestrus^1^.

Items	LD	LE	MD	ME
Raw Reads	57,434,498	58,294,608	56,257,632	57,417,062
Clean Reads (%^2^)	54,936,961	56,007,603	54,394,534	56,015,413
	(95.63%)	(96.09%)	(96.70%)	(97.56%)
Adapter Polluted Reads (%)	1,271,411	1,098,098	905,188	736,364
	(2.24%)	(1.87%)	(1.61%)	(1.29%)
Ns Reads (%)	20,345	22,398	40,996	67,644
	(0.03%)	(0.04%)	(0.08%)	(0.12%)
Low-quality Reads (%)	1,205,779	1,166,507	916,912	597,639
	(2.10%)	(2.00%)	(1.62%)	(1.04%)
Clean Q30 Bases Rate (%)	88.25	87.87	91.65	95.68
Mapped Reads (%)	42,460,339	44,601,507	42,932,065	45,122,743
	(77.33%)	(79.63%)	(78.93%)	(80.55%)
Multi Map Reads (%^3^)	2,407,266	2,658,098	2,547,627	2,531,558
	(4.33%)	(5.00%)	(5.00%)	(4.67%)

^1^LD, Large white gilts at diestrus; LE, Large white gilts at estrus; MD, Mi gilts at diestrus; ME, Mi gilts at estrus.

^2^The number of clean reading frames of total raw reading frames.

^3^The number of all mapped reads out of total clean reads. Q30 represents an error rate of less than 0.001.

### SNP and InDel analysis

We detected approximately 122,804 to 335,295 SNPs and 6,140 to 14,947 InDel in the 11 samples (Table 2). The most frequent SNPs were A>G, C>T, G>A, and T>C (Supplementary Fig. S1). The most frequent length of InDel was one nucleotide (Supplementary Fig. S2).

**Table 2:** Statistics of SNP and InDel of the genes expressed in follicles tissue of Large white and Mi gilts at estrus and diestrus.

Sample^1^		SNP	InDel	Total
LD	NO_1	201,224	11,884	213,108
	NO_2	146,956	8,966	155,922
	NO_3	120,548	7,112	127,660
LE	NO_4	140,845	8,494	149,339
	NO_5	255,190	14,947	270,137
	NO_6	146,954	8,737	155,691
MD	NO_7	335,295	18,467	353,762
	NO_8	122,804	6,140	128,944
ME	NO_9	202,764	11,187	213,951
	NO_10	206,295	11,126	217,421
	NO_11	266,848	14,711	281,559

^1^LD, Large white gilts at diestrus; LE, Large white gilts at estrus; MD, Mi gilts at diestrus; ME, Mi gilts at estrus.

### Alternative splicing analysis

**Figure 1:**
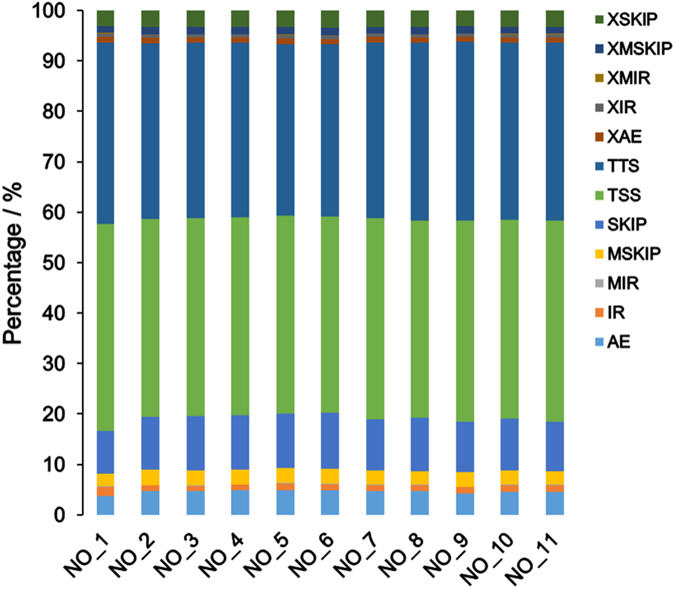
Statistics of alternative splicing events of the mRNA expressed in follicles tissue of Large white and Mi gilts at estrus and diestrus. LD group: NO_1, NO_2, NO_3; LE group: NO_4, NO_5, NO_6; MD group: NO_7, NO_8; ME group: NO_9, NO_10, NO_11. SKIP: Skipped exon; MSKIP: Multi-exon SKIP; IR: Intron retention; MIR: Multi-IR; AE: Alternative exon ends (5’or/and 3’) ; TSS: Transcription Start Site; TTS: Transcription Terminal Site; XSKIP: Approximate SKIP; XMSKIP: Approximate MSKIP; XIR: Approximate IR; XMIR: Approximate MIR; XAE: Approximate AE.

We detected 12 types of alternative splicing (**AS**) events: Skipped exon (SKIP), Multi-exon SKIP (MSKIP), Intron retention (IR), Multi-IR (MIR), Alternative exon ends (5’or/and 3’) (AE), Transcription Start Site (TSS), Transcription Terminal Site (TTS), Approximate SKIP (XSKIP), Approximate MSKIP (XMSKIP), Approximate IR (XIR), Approximate MIR (XMIR), and Approximate AE (XAE). The top two AS events were TSS and TTS AS events, which accounted for more than 70% of AS events in each library (Fig. 1).

### Identification and analysis of DEGs

**Figure 2:**
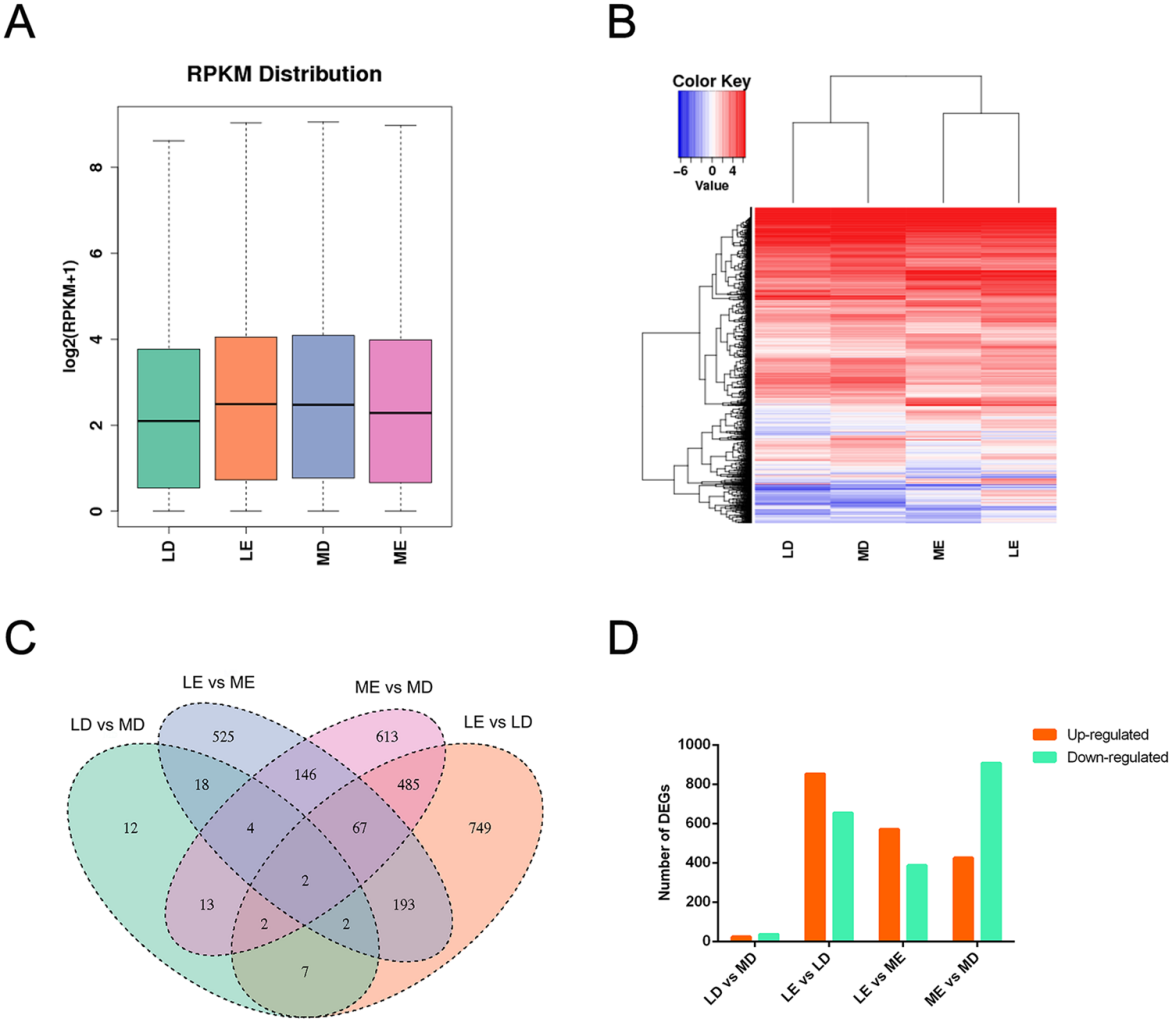
Identification and analysis of DEGs of the genes expressed in follicles tissue of Large white and Mi gilts at estrus and diestrus. LD, Large white gilts at diestrus; LE, Large white gilts at estrus; MD, Mi gilts at diestrus; ME, Mi gilts at estrus. (**A**) Comparison of expression levels of genes detected in the LD, MD, LE, and ME groups. (**B**) Heatmap analysis of DEGs in the LD, MD, LE, and ME groups. (**C**) Venn diagram of the 2838 differentially expressed genes detected among LD vs MD, LE vs ME, LE vs LD, ME vs MD comparison groups. Different color represents different combination, the number in the overlap region represents the overlapped DEGs number. (**D**) Number of enriched DEGs. The orange and cyan pillars represents up-regulated and down-regulated gene number, respectively.

A total of 20,910 genes were expressed in the 11 samples (Supplementary Table S4). The number of expressed genes was not significantly different between libraries. As shown in Table 3, reference genes expressed at RPKM < 10 and RPKM > 500 were 68.03% to 72.81% and 0.75% to 0.78%, respectively. In addition, the gene expression levels between groups were not significantly different (*P* > 0.05, Fig. 2A). A DEG heatmap showed that the DEGs from LD vs MD comparison group gather together, while the DEGs from LE vs ME comparison group gather in another class (Fig. 2B). Subsequently, 2,838 DEGs were identified (Supplementary Table S5) using DESeq (v1.14.0). The distribution of DEGs is revealed in a Venn diagram (Fig. 2C), where tartrate-resistant acid phosphatase type 5 (*ACP5*) and GPI transamidase component PIG-S-like (*PIGS*) were the common DEGs among the four comparison groups. The number of DEGs was much greater (One-Way ANOVA, *P* = 0.018) at estrus than diestrus. The number of DEGs was not different (One-Way ANOVA, *P* = 0.543) between Large White and Mi gilts at estrus or diestrus (Fig. 2D). Twenty-six common DEGs were found in the LD vs MD and LE vs ME comparison groups (Table 4). To screen potential candidate genes associated with estrus expression, the fold change was further analyzed and the *P*-values of up-regulated and down-regulated DEGs estimated (Supplementary Fig. S3). A DEG whose reads count was zero in one of the four groups was defined as a specific expressed gene (Supplementary Table S6). A new gene (ENSSSCG00000028235) was exclusively expressed in Mi gilts, while another new gene (ENSSSCG00000021903) was exclusively expressed in Large White gilts. In addition, the new gene ENSSSCG00000003073 was only expressed at estrus.

**Table 3:** Number of genes within different reads per kilobase transcriptome per million mapped reads (RPKM) intervals.

RPKM	Number of genes^1^ (%)			
	LE	MD	ME	LD
0–10	15,225(72.81%)	14,225(68.03%)	14,536(69.52%)	14,762(70.60%)
10–50	4,213(20.15%)	5,050(24.15%)	4,718(22.56%)	4,532(21.67%)
50–100	749(3.58%)	877(4.19%)	850(4.07%)	825(3.95%)
100–500	566(2.71%)	598(2.86%)	643(3.08%)	628(3.00%)
>500	157(0.75%)	160(0.77%)	163(0.78%)	163(0.78%)

^1^LD, Large white gilts at diestrus; LE, Large white gilts at estrus; MD, Mi gilts at diestrus; ME, Mi gilts at estrus.

**Table 4:** List of 26 overlaped DEGs in LD vs MD and LE vs ME comparison groups^1^.

Gene ID	Log2Fold (LD vs MD)	Log2Fold (LE vs ME)	Up/Down
ENSSSCG00000000047	4.196826985	3.799864112	up
ENSSSCG00000003514	−1.96057002	1.11087342	up
ENSSSCG00000003746	7.164317644	7.951655137	up
ENSSSCG00000007391	5.434905379	2.710809254	up
ENSSSCG00000012517	4.885388402	4.1791834	up
ENSSSCG00000021903	—	—	up
ENSSSCG00000022485	7.3977206	2.517063372	up
ENSSSCG00000022737	—	6.014084639	up
ENSSSCG00000026850	3.835736057	3.802367985	up
ENSSSCG00000026923	—	—	up
ENSSSCG00000027402	−3.344406106	2.258632588	up
ENSSSCG00000029574	6.18186221	3.649117191	up
ENSSSCG00000030790	9.02308197	6.693415446	up
ENSSSCG00000030888	3.482952208	5.527148227	up
ENSSSCG00000000382	−3.920837477	−3.820658642	down
ENSSSCG00000003278	−3.418551101	−3.056977033	down
ENSSSCG00000004181	−3.437416724	−2.163910128	down
ENSSSCG00000007436	−3.592691493	−2.724788739	down
ENSSSCG00000012759	−4.676540398	−4.510439496	down
ENSSSCG00000013612	−2.291163851	−2.212168153	down
ENSSSCG00000021283	−4.112862996	−2.722427296	down
ENSSSCG00000022129	−2.496457265	−1.357534888	down
ENSSSCG00000023383	—	−1.352909641	down
ENSSSCG00000024011	—	−8.719586523	down
ENSSSCG00000028235	—	—	down
ENSSSCG00000029040	—	−7.975214263	down

^1^LD, Large white gilts at diestrus; LE, Large white gilts at estrus; MD, Mi gilts at diestrus; ME, Mi gilts at estrus. “-” represent one or two groups’ reads count were 0.

### GO and KEGG pathway enrichment analysis

**Figure 3:**
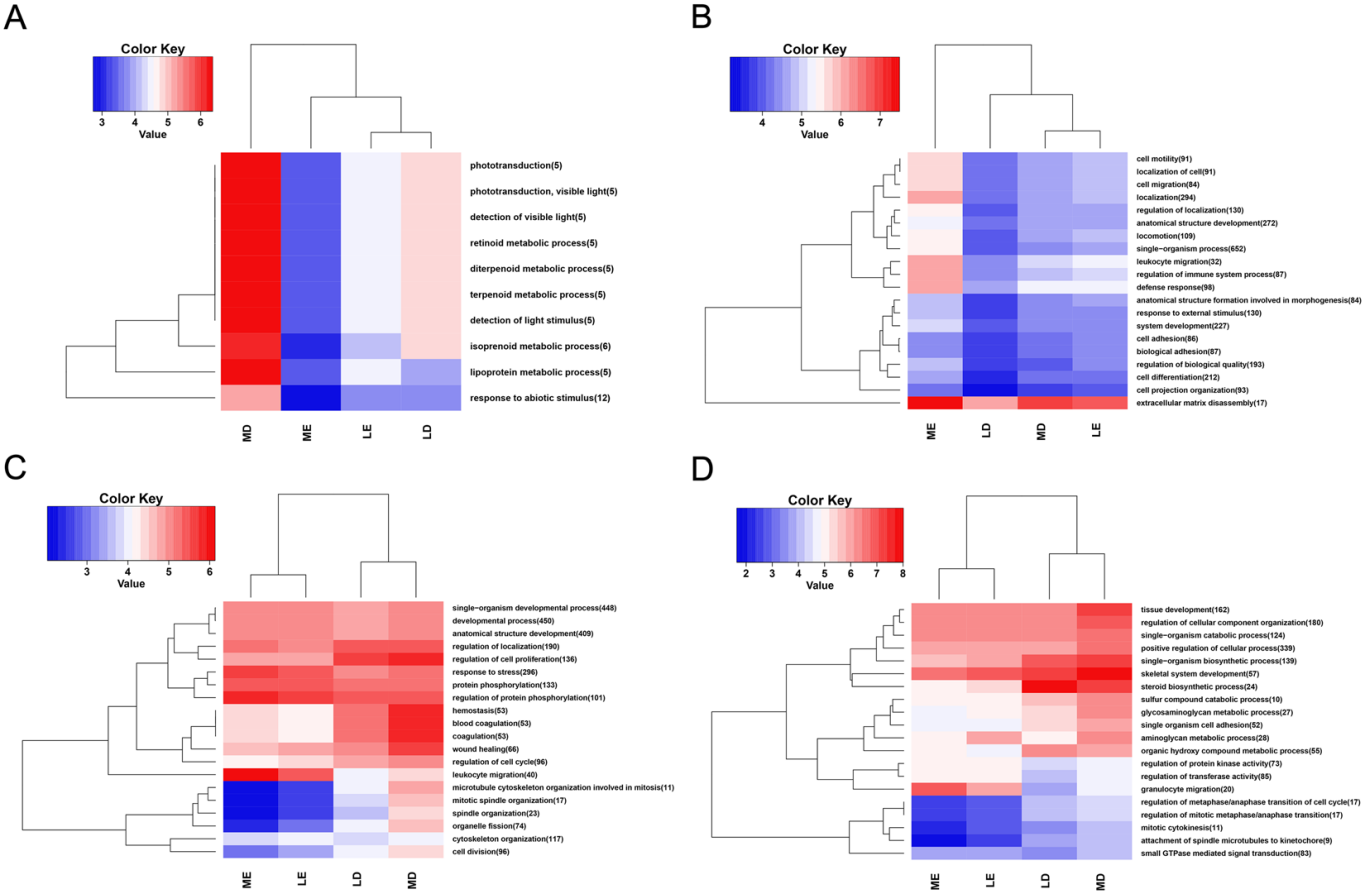
GO annotation of DEGs of the genes expressed in follicles tissue of Large white and Mi gilts at estrus and diestrus. LD, Large white gilts at diestrus; LE, Large white gilts at estrus; MD, Mi gilts at diestrus; ME, Mi gilts at estrus. DEGs number of the most enriched 20 GO terms derived from biological processes from LD vs MD (**A**), LE vs ME (**B**), LE vs LD (**C**), ME vs MD (**D**) comparison groups, respectively. Red shows higher expression and blue shows lower expression. The figures in parentheses refer to the number of DGEs in this term.

The enrichment of DEGs in the GO terms was tested to gain the insights into the biological implications. These DEGs fell into three major GO categories: biological process, cellular component, and molecular function. In the GO category biological process, the most abundant GO terms were single-organism process, cellular process, metabolic process, and biological regulation. In the LD vs MD, LE vs ME, LE vs LD, and ME vs MD comparison groups, the number of biological processes with significant different (q < 0.05) DEGs were 10, 108, 178, and 495, respectively (Fig. 3; Supplementary Table S7). In the GO category cellular component, the most abundant GO terms were cell part and organelle. In the GO category molecular function, the most abundant GO terms were binding and catalytic activity.

**Figure 4:**
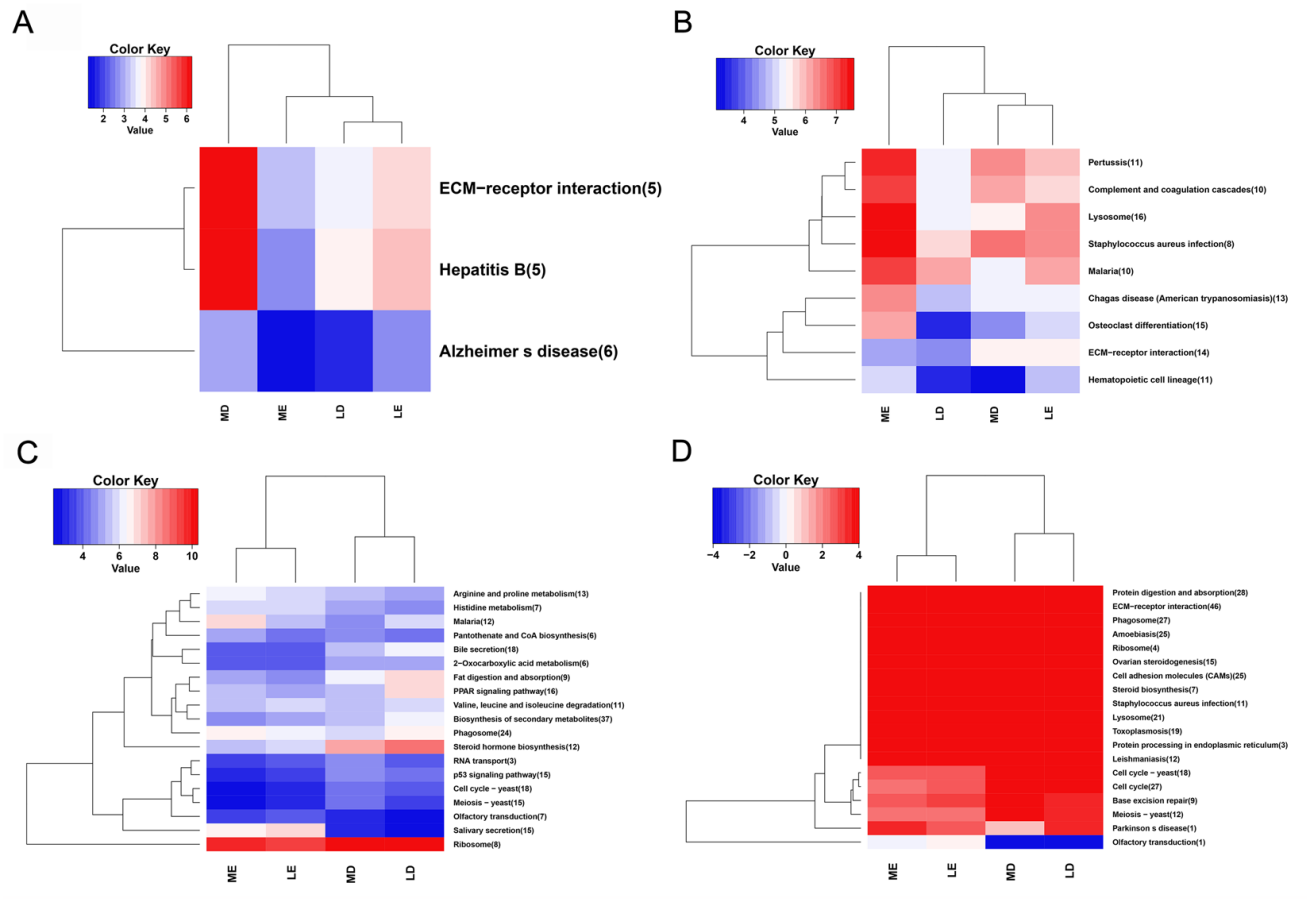
KEGG annotation for DEGs functions of the genes expressed in follicles tissue of Large white and Mi gilts at estrus and diestrus. LD, Large white gilts at diestrus; LE, Large white gilts at estrus; MD, Mi gilts at diestrus; ME, Mi gilts at estrus. DEGs number of the most enriched 20 pathways from LD vs MD (**A**), LE vs ME (**B**), LE vs LD (**C**), ME vs MD (**D**) comparison groups, respectively. Red shows higher expression and blue shows lower expression. The figures in parentheses refer to the number of DGEs in this pathway.

To identify the potential biological pathways involved in estrous behavior, a KEGG analysis was performed. The DEGs were enriched in 315 pathways in the LD vs MD, LE vs ME, LE vs LD, and ME vs MD comparison groups. There were 3, 10, 24, and 23 pathways with significant differences (q < 0.05) of DEGs in the LD vs MD, LE vs ME, LE vs LD, and ME vs MD comparison groups, respectively (Fig. 4; Supplementary Table S8). The representative KEGG pathways, such as ECM−receptor interaction, olfactory transduction, ovarian steroidogenesis, cell cycle, steroid biosynthesis, cell cycle-yeast, phagosome, and ribosome, were enriched with these DEGs. Most DEGs were upregulated in the MD and LE groups. In the ECM−receptor interaction pathway (Table 5), five novel genes were upregulated in the MD group, but none in the LD group. The CD36 molecule (*CD36*) and a novel gene were upregulated in the ME group. Twelve genes, such as collagen type XI alpha 1 chain (*COL11A1*), thrombospondin 1 (*THBS1*), etc., were upregulated in the LE group. Forty genes were upregulated in the MD group, such as laminin subunit beta 2 (*LAMB2*), collagen type VI alpha 5 chain (*COL6A5*), hyaluronan mediated motility receptor (*HMMR*), etc. Six genes, syndecan 1 (*SDC1*), secreted phosphoprotein 1 (*SPP1*), integrin subunit beta 3 (*ITGB3*), etc., were upregulated in the ME group. In the steroid biosynthesis pathway (Table 5), nine genes, such as hydroxysteroid (17-beta) dehydrogenase 7 (*HSD17B7*), 24-dehydrocholesterol reductase (*DHCR24*), methylsterol monooxygenase 1 (*MSMO1*), etc., were upregulated in the LD group. However, no gene was upregulated in the LE group. In addition, six genes, such as *DHCR24*, *MSMO1*, transmembrane 7 superfamily member 2 (*TM7SF2*), etc., were upregulated in the MD group. Lipase A, lysosomal acid type (*LIPA*) was upregulated in the ME group. Fifteen of 23 genes involved in the ovarian steroidogenesis pathway (Table 5), such as low-density lipoprotein receptor (*LDLR*), *CYP19A1*, *HSD3B1*, etc., were upregulated in the LD group. Eight genes, such as arachidonate 5-lipoxygenase (*ALOX5*), phospholipase A2 group IVA (*PLA2G4A*), adenylate cyclase 3 (*ADCY3*), etc., were upregulated in the LE group. In constrast, 14 genes (*LDLR*, *HSD3B1*, *CYP17A1*, etc.) were upregulated in the MD group, while one novel gene was upregulated in the ME group.

**Table 5:** Several representative heat-related pathways in LD vs MD, LE vs ME, LE vs LD, and ME vs MD comparison groups for DEGs.

Group	Pathway	Up_Gene	Down_Gene
LD vs MD	ECM-receptor interaction	—	5 novel genes
LE vs ME	ECM-receptor interaction	COL11A1, THBS1, ITGA11, and 9 novel genes	CD36 and one novel gene
LE vs LD	Ovarian steroidogenesis	PTGS2, ALOX5, PLA2G4A, ADCY3, CYP2J34, ADCY7, and 2 novel genes	LDLR, HSD17B7, CYP19A1, CYP11A1, PRKX, HSD3B1, CYP17A1, SCARB1, HSD17B1, and 6 novel genes
	Steroid biosynthesis	—	CYP51A1, HSD17B7, DHCR24, MSMO1, SC5D, TM7SF2, EBP, and 2 novel genes
	Steroid hormone biosynthesis	UGT2A3, CYP7A1, and one novel gene	HSD17B7, CYP19A1, CYP11A1, HSD3B1, CYP17A1, HSD17B1, CYP21A2, and 2 novel genes
ME vs MD	ECM-receptor interaction	CD36, SDC1, CD44, SPP1, ITGB3, and one novel gene	LAMB2, COL6A5, COL5A1, COL4A, LAMC3, COL6A2, ITGA6, COL11A1, COL6A3, HMMR, COL2A1, LAMC1, DAG1, and 27 novel genes
	Ovarian steroidogenesis	PTGS2	LDLR, CYP19A1, HSD3B1, CYP17A1, SCARB1, HSD17B1, and 8 novel genes
	Steroid biosynthesis	LIPA	DHCR24, MSMO1, TM7SF2, EBP, and 2 novel genes

LD, Large white gilts at diestrus; LE, Large white gilts at estrus; MD, Mi gilts at diestrus; ME, Mi gilts at estrus.

### qRT-PCR confirmation

To validate the results of RNA-seq, *SULT1C3*, apolipoprotein E (*APOE*), complement component C7 precursor (*C7*), *CYP17A1*, complement component 4 binding protein, alpha (*C4BPA*), gap junction protein alpha 1 (*GJA1*), and *ACP5* were selected for quantitative PCR (qPCR). Based on qRT-PCR, these 7 DEGs were differently expressed in at least one of four comparison groups. Some DEGs have been reported to be involved in follicular development processes. For example, *ACP5* was differentially expressed in the four groups; *APOE*, *C4BPA*, *C7*, and *ACP5* were highly expressed at estrus; *SULT1C3*, *CYP17A1*, and *GJA1* were highly expressed at diestrus. In addition, it has been reported that *SULT1C3* ^28^ and *CYP17A1* ^29^ are related to steroid metabolism pathway, *APOE* was related to lipid metabolism^30^, and *C7* ^31^ and *C4BPA* ^32^ were related to the immune system. The expression patterns of these 7 DEGs were in agreement with our RNA-seq findings. As shown in Table 6, the correlation coefficients between qRT-PCR and RNA-seq results are greater than 0.9 6 of 7 DEGs; the P value are less than 0.05 in 4 DEGs and less than 0.10 in 6 of 7 DEGs. For C7 gene, the correlation coefficient between results of qRT-PCR and RNA-seq is 0.86 (*P* = 0.14). The qRT-PCR results were consistent with the sequencing data (shown in Supplementary Fig. S4). Although the relative expression of C7 in LE vs LD and GJA1 LE vs ME comparison groups differed using two approaches, the difference was not significant, which was probably caused by biological differences between samples as well as the sensitivity and capability of the different methods.

**Table 6:** Validation of RNA-seq results using quantitative RT-PCR.

Gene ID	Gene name	Item	LD	LE	MD	ME	R	*P* value
ENSSSCG00000003088	APOE	RNA-seq	318.74	1868.87	601.92	9636.59	0.92	0.08
		QRT-PCR	0.06	0.64	0.11	1.1		
ENSSSCG00000016859	C7	RNA-seq	189.43	226.24	73.21	127.66	0.86	0.14
		QRT-PCR	1.42	1.27	0.5	1.14		
ENSSSCG00000010591	CYP17A1	RNA-seq	425	9.17	591.97	12.94	0.95	0.05
		QRT-PCR	19.08	0.94	44.42	2.75		
ENSSSCG00000004241	GJA1	RNA-seq	356.47	207.98	425.79	124.65	0.93	0.07
		QRT-PCR	1.94	0.84	2.53	1.05		
ENSSSCG00000015663	C4BPA	RNA-seq	27.61	35.16	96.77	286.72	0.98	0.02
		QRT-PCR	0.21	0.24	0.26	1.16		
ENSSSCG00000028691	SULT1C3	RNA-seq	234.52	11.91	9.16	2.85	0.98	0.02
		QRT-PCR	15.15	4.77	2.06	1.44		
ENSSSCG00000013612	ACP5	RNA-seq	6.84	61.37	39.48	261.79	0.99	0.01
		QRT-PCR	0.06	0.4	0.15	1.2		

LD, Large white gilts at diestrus; LE, Large white gilts at estrus; MD, Mi gilts at diestrus; ME, Mi gilts at estrus. The RNA-seq data for the DEGs represent the RPKM values in the four groups; Relative expression levels of the selected target genes were calculated with the 2(-Delta Delta C[T]) method The QRT-PCR data represent the relative expression levels of DEGs, the relative expression levels of the four groups were relative to the ME group.

## Discussion

In recent years, high-throughput sequencing have been performed using animal reproductive tissues, such as endometrium^20^, ovaries^21^, placenta^33^, follicles^34^ and granulosa cells^35^, to identify the genes and genetic loci that affect litter size. Compared to litter size, the studies on the molecular genetic mechanism of estrus expression have seldom been reported. In commercial pig production, estrus characteristics influence sow fecundity^36^. Previous studies have investigated the expression profiles in ovaries of sheep at estrous and anestrous^37^, in porcine endometrial tissue on 9^38^, 12^39^, 14^22^, and 15^38^ d of pregnancy, and in porcine endometrial tissue on 12^39^ d after onset of estrus. In the present study, we investigated the expression profiles in follicle tissue of Large White and Mi gilts at diestrus and estrus using RNA-seq, to mine the DEGs between two breeds and two stages of estrus cycle.

In our present study, 610 million clean reads were generated. In each group, the clean reads were more than 54 million, which is similar to the previous studies on RNA-seq in placenta^33^ and longissimus muscle^40^. A total of 122,804–335,295 SNPs and 6,140-14,947 InDel were detected in the 11 samples. The number of SNPs and InDel were much greater for Mi gilts than Large White gilts at both estrus and diestrus stages, which indicates the difference on estrus expression between two breeds was due to the difference of gene expression. In addition, the most SNPs (A>G, C>T, G>A, and T>C) and the most length of InDel (one nucleotide) could play important roles in the regulation of gene expression^41^. AS generates multiple transcripts from the same gene, and widely exists in eukaryotes^42^. AS is an important way to regulate gene expression and protein diversity^43^. The most common AS events (TSS and TTS) can produce different transcripts or different proteins which affect the biological process^43^.

A total of 2,838 DEGs produced in the LD vs MD, LE vs ME, LE vs LD, and ME vs MD comparison groups were annotated in the GO and KEGG databases in the present study. Some DEGs had been identified in follicle development and hormone metabolism by RNA-seq^37, 44^. Further validations had been made using quantitative PCR for a part of DEGs, such as insulin-like 3 precursor (*INSL3*), *SULT1C3*, and hypoxia-inducible factor 1-alpha isoform 3 (*HIF1ß*)^20, 21, 37^. Previous studies suggested that *INSL3* might be an important intrafollicular modulator of theca interna cell function/steroidogenesis^45^ and theca cell-derived growth factor for pre antral follicle^46^. It has been reported that *SULT1C3*, a member of sulfotransferase family, catalyzes the sulfate conjugation of several hormones^47, 48^, and regulates estrogen removal in estrogen metabolic pathway^19^. In addition, *HIF1ß* is a critical mediator of follicle development and ovulation and a regulator of gene expression in ovary^49, 50^. Several genes, such as *INSL3*, *STAR*, and *HIF1ß*, were associated with follicular development, ovulation, and steroid secretion in the ovary of Qira black sheep at estrous and anestrous^37^. *VEGF* and *STAR* play important roles in follicle development and steroid synthesis in Qira Black Sheep and Hetian Sheep^21^. Angiogenesis is critical for female ovulatory cycle, including follicular development, ovulation, and corpora lutea formation^51^. The VEGF system is the most important signaling pathway in angiogenesis^52^. *STAR* is essential for steroid hormone synthesis and involved in the regulation of follicular development in mammalian ovary^53–55^. But we did not detect *VEGF* and *STAR* in the DEGs, which could be due to the difference between different species. In our present study, there were 26 DEGs between breeds at both diestrus and estrus, which may be caused by the differences between breeds^8^. These 26 DEGs were mainly involved in the single-organism process, and mainly related to binding and catalytic activity. The predicted novel genes in our present study also provided clues for the research on estrus expression between different breeds and different stages of estrus cycle. The function of these new genes has not been identified. Further studies are needed to evaluate if whether they are new candidate genes related to estrus expression.

Follicles, as important tissues of animal reproductive organs, fulfill many pivotal functions, including oocyte and granulosa cells production and hormone secretion. They have obvious differences in shape and biological activity during estrous cycle^21, 37, 56^. In the ovarian follicles, sex steroids are synthesized from granulosa and thecal cells in the follicular wall^34^. Previous study found that two signaling networks (Notch, SLIT/ROBO and PI3K signaling, and ITGB5 and extracellular matrix signaling) were associated with follicular development through extracellular signal related kinases (ERKs)^57^. In our present study, the GO annotation and KEGG pathway analysis clearly revealed that some hormone related genes were involved in steroid biosynthesis and ovarian steroidogenesis pathways, which indicates that the two pathways were activated in the ovaries of Large White and Mi gilts at estrus. In the steroid biosynthesis and ovarian steroidogenesis pathway, most of the DEGs were upregulated in the LD and MD groups, which suggests that these genes were activated and overexpressed during the diestrus. Most of these genes belong to cytochrome P450 family and oxidoreductases family, and participate in the redox reaction^19^. *SCARB1* and *LDLR* have been shown to be involved in biosynthesis of steroid hormones through affecting on the absorption of cholesterol substrates from circulating lipoproteins^58–60^, which suggests that lipid metabolism also play a role at estrus. Other genes related to steroid hormones could also be important to ovarian function in regulation of female reproduction.

In addition, follicular development also integrates the proliferation and differentiation of cells in the ovarian follicular wall with the transportation of nutrients, and the supplementation of energy^61, 62^. Results of our present study provided abundant information for some signaling pathways, such as ECM–receptor interaction, olfactory transduction, cell cycle, cell cycle-yeast, phagosome, ribosome, PI3K-Akt signaling, metabolic pathways, DNA replication, cell adhesion molecules (CAMs), and biosynthesis of secondary metabolites. Results of our present study indicates that these pathways potentially regulate estrus expression. Among these signaling pathways, ECM-receptor interaction and olfactory transduction had significant difference in three of four comparison groups. The extracellular matrix (ECM) consists of a complex mixture of structural and functional macromolecules, and plays an important role in tissue and organ morphogenesis^63^. Specific interaction between cells and the ECM leads to the direct or indirect control of cellular activities, such as adhesion, migration, differentiation, proliferation, and apoptosis^64^. In addition, a previous study performed a genome-wide analysis of domestic pigs and Tibetan wild boars, and found that 35 genes were enriched in olfactory transduction pathway^65^. It is a better response to a wider range of food types because of resource-poor plateau^65^. Moreover, a better sense of smell may also be favored by artificial selection because it enhances disease aversion and facilitates the estrus expression and ovulation of females, which is stimulated by the pheromones in the saliva of boars and then enhances sexual receptive behavior of females^66, 67^. In the present study, most DEGs in the pathways affected by different stages of estrus within breeds were activated and overexpressed at estrus. These pathways also have important roles in modulating the balance of integrating cell proliferation, energy supplementation, nutrient delivery, and steroid hormone synthesis, which are important processes for sustaining normal folliculogenesis^34^ and estrus.

In addition, 16 genes involved in cell adhesion molecules (CAMs) were also downregulated at estrus. CAMs, as cell surface proteins, were involved in many biological processes, such as cell adhesion, proliferation, differentiation, developmental processes of fertilization, embryogenesis, and embryonic development^68, 69^. During the rapid growth and development of follicles, a large amount of energy is required to supply ATP for DNA and protein synthesis in the follicular membrane^70^. Cell cycle, cell cycle-yeast, ribosome, DNA replication, biosynthesis of secondary metabolites and metabolic pathways are related to the growth and development of the follicle^34^, as well as expression of estrus. In our present study, most of the DEGs involved in metabolic pathways (91/135) and cell cycle (26/27) were downregulated at estrus, which indicates that these DGEs involved in metabolic pathways and cell cycle pathways could inhibit the follicular development, and ovulation. However, the exact relationships between these signaling networks are not fully understood, and no evidences has been reported to support the effects of these pathways at estrus. In our present study, some DEGs repeatedly appeared in various signaling pathways, and their roles at estrus should be studied in the future.

In conclusion, we generated the expression profile of mRNA in pig follicles of Large White gilts at diestrus (LD) and estrus (LE), Mi gilts at diestrus (MD) and estrus (ME) using RNA-seq. A total of 122,804-335,295 SNPs, 6,140-14,947 InDel and 12 types of AS events (39.57% TSS, 34.90% TTS) were detected in the 11 samples. A total of 2,838 genes were differentially expressed between the groups. These DEGs are involved in important biological processes, such as single-organism process, binding, catalytic activity, cell adhesion and enriched in ECM-receptor interaction, olfactory transduction, ovarian steroidogenesis, cell cycle, steroid biosynthesis, CAMs, phagosome, and ribosome signaling pathways, which depicts a complex mechanism underlying the modulation of estrus characteristics. Thus, these newly identified DEGs should be considered as new candidate genes with functions in regulating estrus. These results provide useful information for future in-depth studies on estrus expression.

## Materials and Methods

### Ethics Statement

All experimental procedures were conducted according to the “Guidelines for Experimental Animals” of the Ministry of Science and Technology (Beijing, China). This study was reviewed and approved by the ethics committee of Nanjing Agricultural University. Animals were humanely sacrificed as necessary to ameliorate suffering.

### Animals

Thirty Large White gilts and 30 Mi gilts were observed for expression of estrus at Yong Kang Agricultural Science and Technology Co., Ltd in Changzhou city, Jiangsu Province, China. The gilts were selected before their second estrus cycle. Their breed, group, body weight, and backfat thickness information are shown in Supplementary Table S1. The Large White and Mi gilts were raised in under a standardized feeding regimen with free access to water.

### Estrus detection and sample collection

Estrus detection was carefully performed twice daily (at 7:00 and 15:00) at pro-estrus. Expression of estrus were defined as standing reflex, and reddening and swelling of the vulva^71^. Inspections to evaluate standing reflex, and reddening and swelling of the vulva were made on gilts individually by an experienced technician using a standardized routine^3^. A sexual mature boar was introduced into each pen for at least 15 min daily to ensure adequate stimulation, during which the gilts had directly head-to-head contact with the boar. Standing reflex was assessed by the back-pressure test. Reddening and swelling of the vulva was measured visually. When a standing reflex occurred, the expression of estrus were scored (0: no; 1: weak; 2: strong)^13, 36, 72^ according to a standard scoring system (Table 7).

**Table 7:** Scoring systems for estrus expression of Large white and Mi gilts.

Estrus expression		Standing reflex	Reddening and swelling of the vulva	Mucus discharge from the vulva
Scoring standards	0	No standing reflex for the back-pressure test within 5 min, or weakly exhibits the standing reflex for the back-pressure test after 5 min	White, or no difference with skin color, no swelling	No mucus
	1	Moderately exhibits the standing reflex after the back-pressure test (stands for boar but moves around some) within 5 min	Pink, slightly heavier than the skin color, slight swelling, a little fold	A small amount of mucus, pussy humid, can’t be pulled into filamentous
	2	Strongly exhibits the standing reflex after the back-pressure test (stands firm without any movement)	Red to fuchsia, significantly deeper than the skin color, swelling significantly, no fold, present bullate	Mucus presents transparent to yellow and viscous, can be formed mucus drops in the corner below the vulva, and mucus can be pulled into filamentous

**Figure 5:**
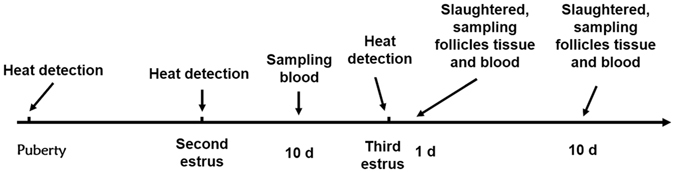
The sampling flow chart.

After the first scoring, Large White and Mi gilts with scores of 2 points were selected (sampling flow chart is shown in Fig. 5). At the onset of standing reflex in the second estrus cycle, amount of 5 ml blood was collected from the anterior vena cava of gilts at the 10th day of estrus cycle. Blood samples were centrifuged at 3,000 rpm for 10 min, immediately after collection. The plasma was stored at −20 °C until analyses. Before the third estrus cycle, 6 Large White gilts and 6 Mi gilts were selected. The estrus cycles were about 21 d in length and the scores of the estrus expression were two points. At the first day of the third estrous, the day the gilts were at onset of exhibiting standing reflex, three Large White and three Mi gilts were slaughtered humanely by anesthesia. At the 10th day of estrous cycle, 3 Large White and 3 Mi gilts were slaughtered in the same way. Ovaries were dissected and all samples were collected with better ovulation points or preovulatory follicles on the surfaces of the ovaries. All samples were immediately frozen in liquid nitrogen and stored at −80 °C until RNA was isolated.

### Total RNA isolation

Total RNA was extracted from the follicles using TRIzol reagent (Invitrogen) and treated with DNase I following the manufacturer’s instructions. Degradation and contamination of total RNA were monitored on 1% agarose gels. RNA concentration was measured using Qubit RNA Assay Kit in Qubit 2.0 Flurometer (Life Technologies, CA, USA), its purity was assessed using the NanoPhotometer® Spectrophotometer (IMPLEN, CA, USA), the concentration and integrity were assessed using a RNA Nano 6000 Assay Kit of the Agilent Bioanalyzer 2100 system (Agilent Technologies, CA, USA).

### Library preparation for RNA-seq

According to the manufacturer’s manual, sequencing libraries were generated using NEBNext Ultra™ RNA Library Prep Kit for Illumina (NEB, USA). Amount of 3 μg RNA per sample was used to purify mRNA using poly-T oligo attached magnetic beads and then the purified mRNA was interrupted into short fragments (about 200 bp) by the fragmentation buffer. First strand cDNA was synthesized using random hexamer primer and M-MuLV Reverse Transcriptase (RNase H). After adding buffer, dNTPs, DNA polymerase I (New England Biolabs) and RNase H (Invitrogen), the second strand cDNA synthesis was subsequently performed using DNA polymerase I and RNase H. After adenylation of 3’ ends of DNA fragments, NEBNext Adapter was ligated to prepare for hybridization. The cDNA fragments with 150–200 bp in length were selected for PCR amplification to create cDNA libraries. The library preparations were sequenced on an Illumina Hiseq 2500 platform and generated 125 bp paired-end reads.

### Data analysis

Raw data sequenced from the last step were saved as a format of fastq. All clean reads were obtained by rejecting low quality sequences or reads (it has more than 5% unknown nucleotides) and sequencing adapters. In addition, the Q20, Q30, and GC content were calculated. The reference genomes and the annotation file were downloaded from ENSEMBL database (http://www.ensembl.org/index.html). The software Bowtie2 (v2.2.3) was used for building the genome index. Clean data were mapped to the reference genome using TopHat (v2.0.12). More than 2 bp of mismatches were not considered. Unmapped or multi-position matched reads were excluded from further analyses. In addition, sequence saturation analyses of the four libraries were executed to provide an overview of the project. Reads count of each gene was counted using HTSeq (v0.6.0). RPKMs were then calculated to estimate the expression level of genes, which can eliminate the effect of sequencing depth and gene length on gene expression levels and the data could be compared directly^27^.

### Identification of SNP, InDel, and Alternative splicing (AS)

SNP and InDel analyses were performed by mapping RNA-seq reads into the genomic of Sus scrofa (sscrofa10.2) using Samtools (v1.3.1)^73^. The AS events for each library using ASprofile.b (v1.0.4).

### Identification of differentially expressed genes (DEGs)

The software DESeq (v1.16) was used for differential gene expression analysis between two samples with biological replicates using a model based on the negative binomial distribution. The *P*-value had been assigned to each gene and adjusted by the Benjamini and Hochberg’s approach for controlling the false discovery rate^74^. Genes with q ≤ 0.05 and |log2_ratio| ≥ 1 were identified as differentially expressed genes (DEGs).

### GO and KEGG enrichment analysis

The GO (Gene Ontology, http://geneontology.org/) enrichment of DEGs was implemented by the hypergeometric test^75^, in which *P*-value was calculated and adjusted as q-value, and data background was genes in the whole genome. GO terms with q < 0.05 were considered to be significantly enriched. GO enrichment analysis can exhibit the biological functions of the DEGs. KEGG (Kyoto Encyclopedia of Genes and Genomes, http://www.kegg.jp/) is a database resource containing a collection of manually drawn pathway maps representing our knowledge on the molecular interaction and reaction networks^76^. The KEGG enrichment of DEGs was also implemented by the hypergeometric test, in which p-value was adjusted by multiple comparisons as q-value. KEGG terms with q < 0.05 were considered to be significantly enriched.

### Real-time RT-PCR

RNA samples from the 11 gilts in the RNA-seq experiment were used to validate the results by quantitative real-time RT-PCR (qPCR), which was performed as previously described^77^. Total cDNA was synthesized using reverse transcriptase Kit (TaKaRa, Dalian). To amplify specific fragments referring to selected regulated genes, specific primers were designed with NCBI Primer-BLAST (Supplementary Table S2). Real-time PCR reactions were performed as described by the manufacturer in triplicate with Maxima^TM^ SYBR Green/ROX qPCR Master Mix (Fermentas) on an Applied Biosystems Step One Plus system using the following program: 10 min at 95 °C; followed by 40 cycles of 15 s at 95 °C and 60 s at 60 °C; then a 55–95 °C melting curve detection. The *GAPDH* gene was used as a control in the experiments. All amplifications were followed by dissociation curve analysis of the amplified products. Relative expression levels of the selected target genes were calculated with the 2(-Delta Delta C[T]) method^78^. The statistical difference in gene expression between groups was analyzed by IBM SPSS Statistics (v20.0). The results are presented as mean ± standard deviation. The correlation between the results of RNA-seq and qPCR was calculated using correlation test (pearson correlation) by IBM SPSS Statistics (v20.0)^38^.

## Electronic supplementary material

Supplementary Figure 1-4

Table S4

Table S5

Table S6

Table S7

Table S8
