# Supplementary material for: Genome-wide differential mRNA expression profiles in follicles of two breeds and at two stages of estrus cycle of gilts
Source: Sci Rep. 2017 Jul 11;7:5052. doi: 10.1038/s41598-017-04336-x (PMC5506030; doi:10.1038/s41598-017-04336-x)
Supplement: Supplementary file 1 — Supplementary Figure 1-4 [file 41598_2017_4336_MOESM1_ESM.doc]

**Supplementary Information for:**

**Genome-wide differential mRNA expression profiles in follicles of two breeds and at two stages of estrus cycle of gilts**

Qingpo Chu^1^, Bo Zhou^1^, Feilong Xu^1^, Ruonan Chen^1^, Chunyan Shen^1^, Tingting Liang^1^, Yuan Li^1^ & Allan P. Schinckel^2^

^1^Department of Animal Genetics, Breeding and Reproduction, College of Animal Science and Technology, Nanjing Agricultural University, Nanjing, 210095, P.R. China.

^2^Department of Animal Sciences, Purdue University, West Lafayette, IN 47907-2054, USA. Correspondence and requests for materials should be addressed to B.Z. (email: [zhoubo@njau.edu.cn](mailto:zhoubo@njau.edu.cn)).

**Supplementary Table S1.** The conditions of Large white and Mi gilts used in the experiments

| Breed | Period | Sample | Group | Body weight /kg | Backfat thickness /mm |
| --- | --- | --- | --- | --- | --- |
| Large white | Diestrus | NO_1 | LD | 120 | 10.7 |
| Large white | Diestrus | NO_2 | LD | 124 | 11.1 |
| Large white | Diestrus | NO_3 | LD | 122 | 15 |
| Large white | Estrus | NO_4 | LE | 110.5 | 15.3 |
| Large white | Estrus | NO_5 | LE | 117.6 | 11.7 |
| Large white | Estrus | NO_6 | LE | 120 | 11.2 |
| Mi gilt | Diestrus | NO_7 | MD | 53.3 | 23.3 |
| Mi gilt | Diestrus | NO_8 | MD | 60.6 | 21.5 |
| Mi gilt | Estrus | NO_9 | ME | 69.2 | 26.1 |
| Mi gilt | Estrus | NO_10 | ME | 74.5 | 18.2 |
| Mi gilt | Estrus | NO_11 | ME | 69.1 | 19.7 |

**Supplementary** Table S2. Sequence of primers for the genes selected for real-time RT-PCR.

| Gene ID | Gene name | Primer sequences (5’-3’) | Product size (bp) |
| --- | --- | --- | --- |
| ENSSSCG00000028691 | SULT1C3 | F:GCCCAGCAAACACCTATCCT | 276 |
|  |  | R:TTCCGGCCCTGTGTTAATCC |  |
| ENSSSCG00000003088 | APOE | F:ATGAGGGTTCTGTGGGTTG | 192 |
|  |  | R:CACTTGGTCAGACAGGGACT |  |
| ENSSSCG00000016859 | C7 | F:TGGAGCATCTTCGGTTGCTT | 192 |
|  |  | R:CACATCTGGCAACAGGGTCT |  |
| ENSSSCG00000010591 | [CYP17A1](http://www.ensembl.org/Sus_scrofa/Gene/Summary?g=ENSSSCG00000010591&db=core) | F:ATTGACTCCAGCATTGGCGA | 179 |
|  |  | R:CCGAAGGGCAAGTAGCTCAA |  |
| ENSSSCG00000015663 | [C4BPA](http://asia.ensembl.org/Sus_scrofa/Gene/Summary?g=ENSSSCG00000015663&db=core) | F:GTTTCTCCTGCACTGCACAC | 183 |
|  |  | R:GACCAGCGTGAAGAACGACA |  |
| ENSSSCG00000004241 | [GJA1](http://www.ensembl.org/Sus_scrofa/Gene/Summary?g=ENSSSCG00000004241&db=core) | F:GGTGTCTCTCGCCTTGAACA | 126 |
|  |  | R:CAGTCTTTGGAGGGGCTCAG |  |
| ENSSSCG00000013612 | ACP5 | F:TATCCTGTGTGGTCCATCGC | 185 |
|  |  | R:GAGGGGTCCATGAAGTTCCC |  |
| ENSSSCG00000000694 | GAPDH | F:CCTTCATTGACCTCCACTACATGGT | 161 |
|  |  | R:CCACAACATACGTAGCACCACGAT |  |

**Supplementary Table S3.** The genic distribution of reads information in four groups.

| Group | Exon | Intron | Intergenic |
| --- | --- | --- | --- |
| LD | 18071752(67.09%) | 2298182(8.53%) | 6566432(24.38%) |
| LE | 17774706(67.08%) | 2009569(7.58%) | 6714707(25.34%) |
| MD | 16812798(68.56%) | 1714715(6.99%) | 5993969(24.44%) |
| ME | 18139533(65.26%) | 2459472(8.85%) | 7198345(25.90%) |
| Total | 70798788(66.95%) | 8481938(8.02%) | 26473453(25.03%) |


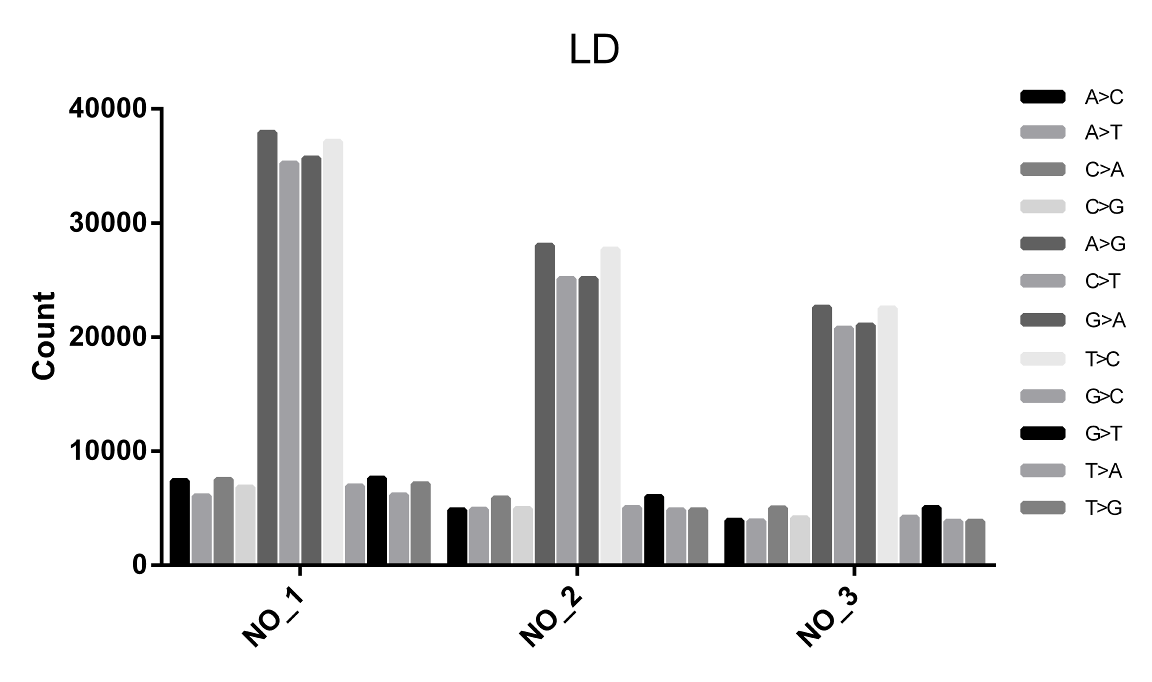

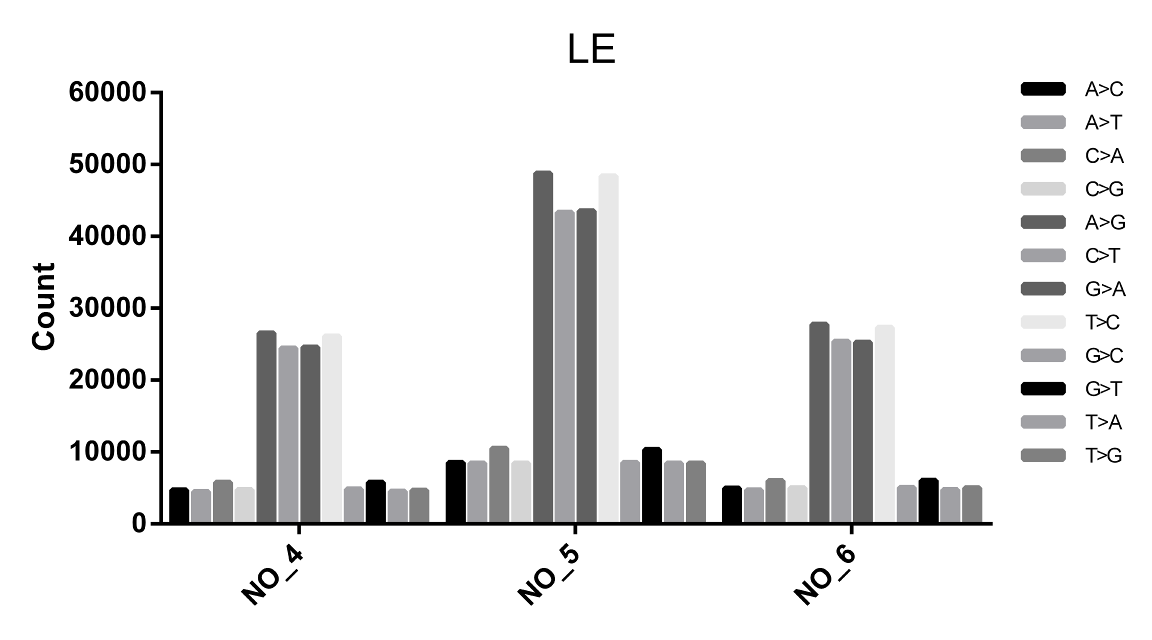

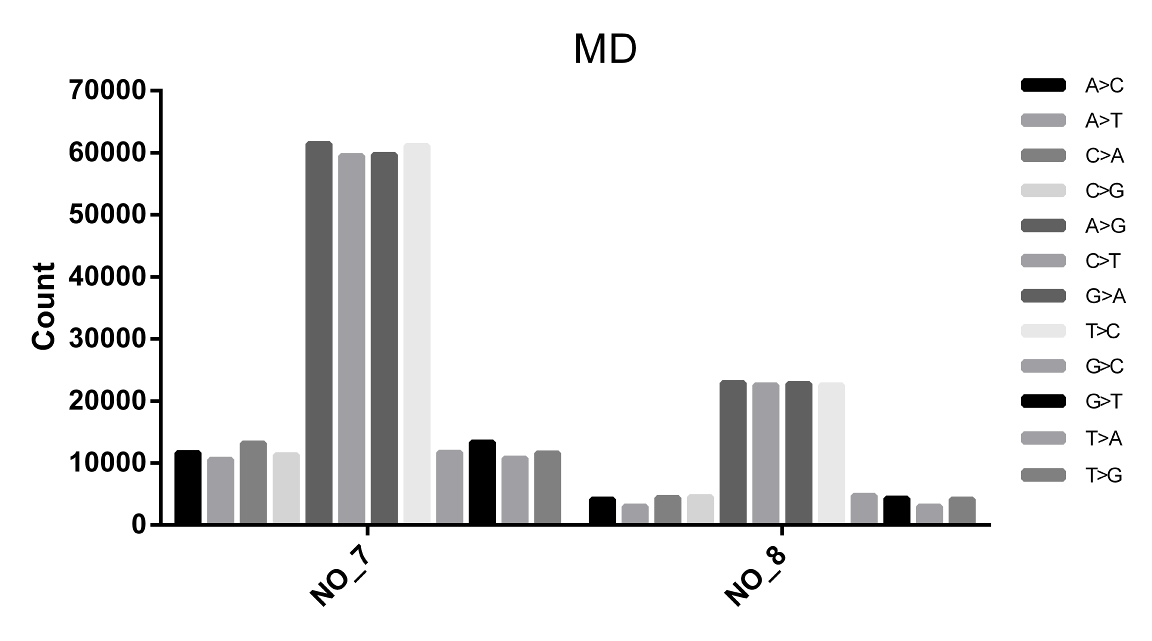

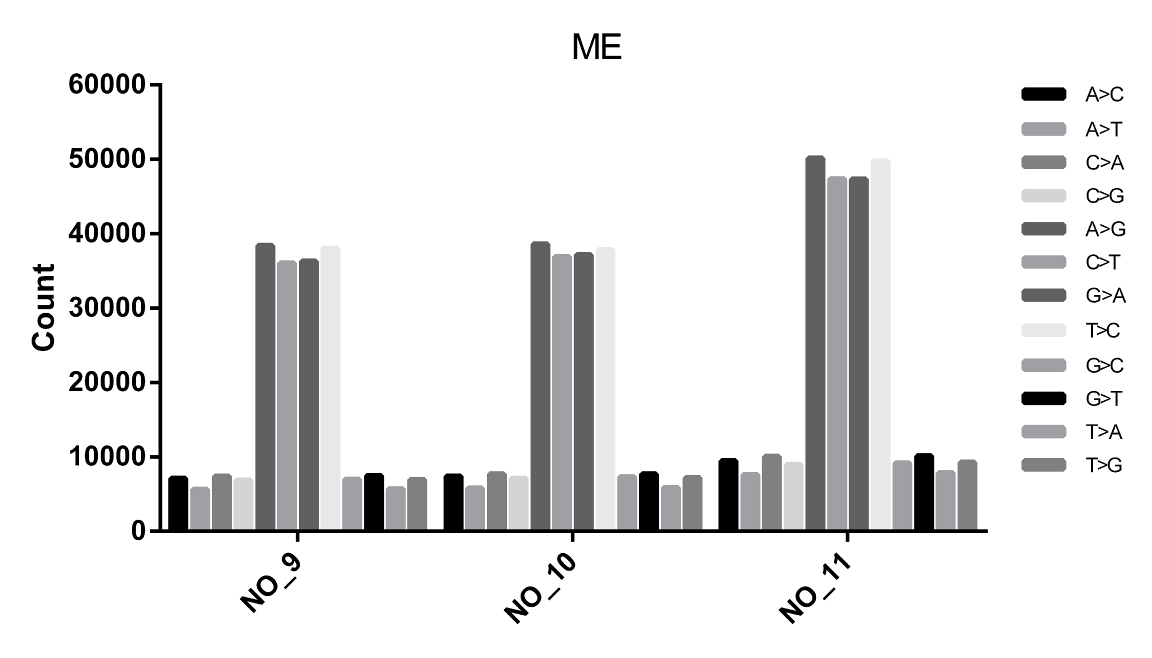


**Supplementary Figure S1. The number of SNP in 11 samples.** LD group: NO_1, NO_2, NO_3; LE group: NO_4, NO_5, NO_6; MD group: NO_7, NO_8; ME group: NO_9, NO_10, NO_11.


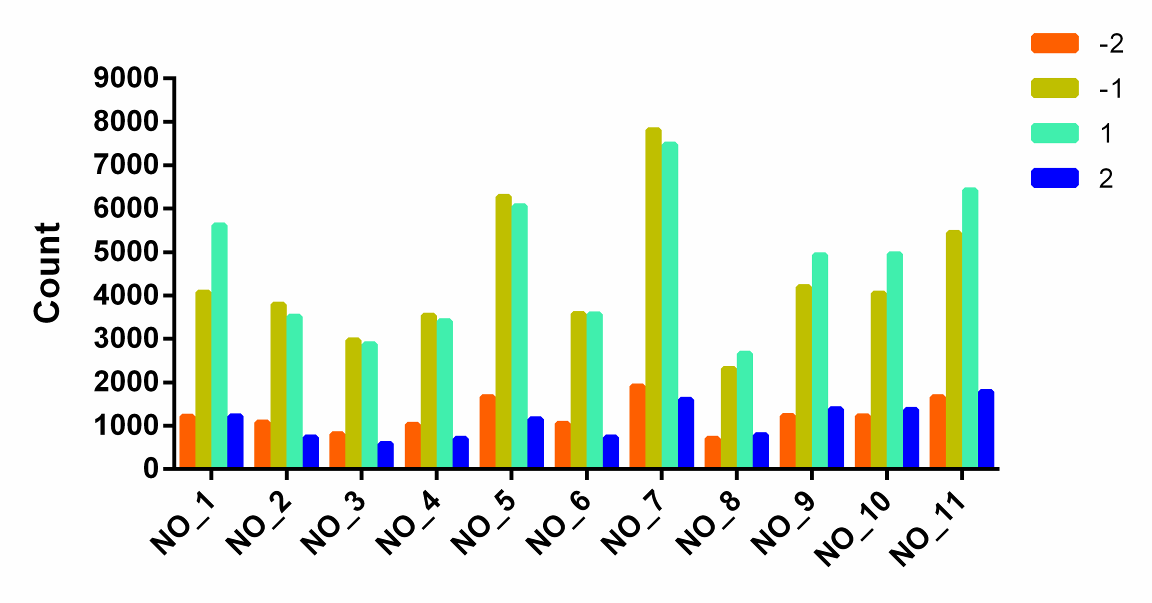


**Supplementary Figure S2. The length of InDel in 11 samples.** LD group: NO_1, NO_2, NO_3; LE group: NO_4, NO_5, NO_6; MD group: NO_7, NO_8; ME group: NO_9, NO_10, NO_11.

**
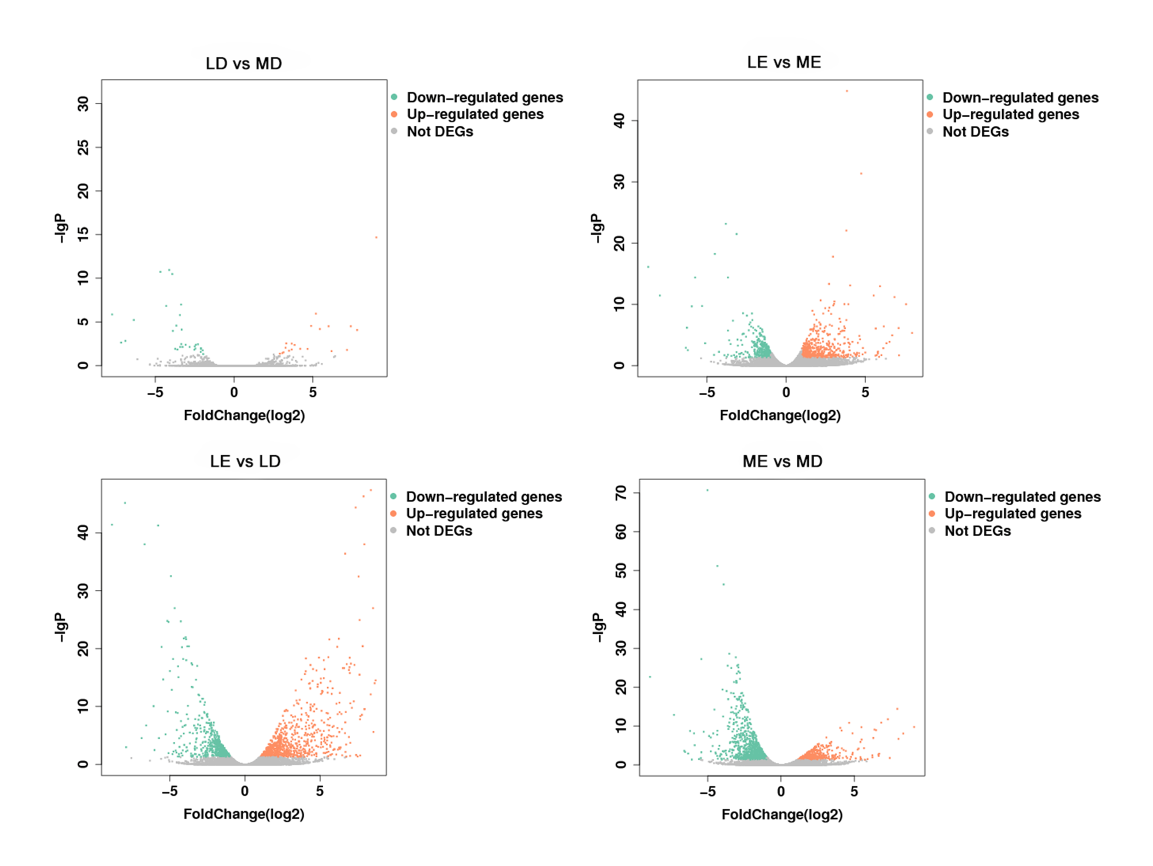
**

**Supplementary Figure S3. Comparison of gene expression levels in the LD vs MD, LE vs ME, LE vs LD, ME vs MD comparison groups.** The expression levels are estimated by RPKM value. The x-axis shows the fold change in different experimental groups, and the y-axis shows the statistically significant degree of change in expression level.


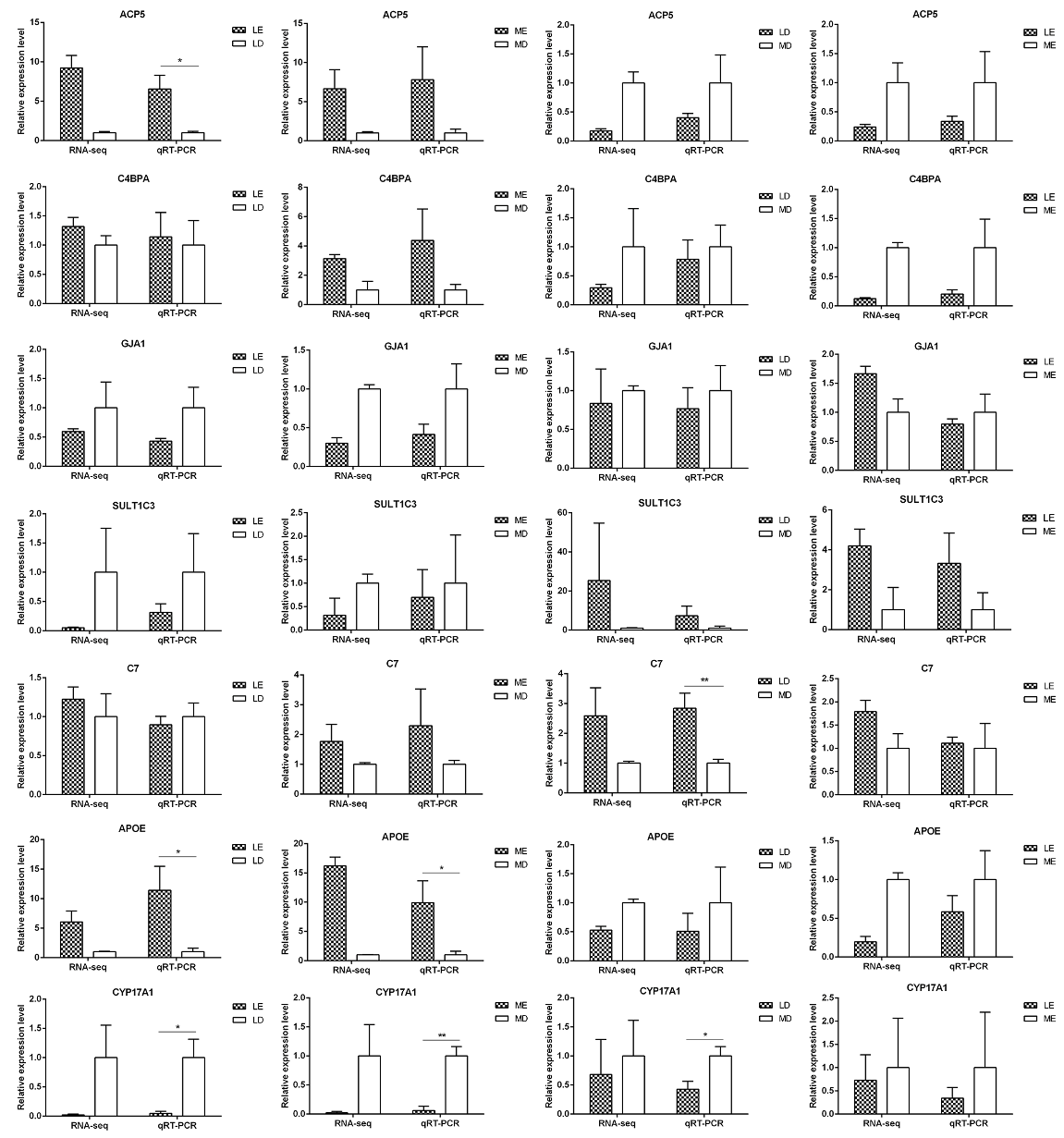


**Supplementary Figure S4. Validation of DEGs by real-time PCR.** LD, Large white gilts at diestrus; LE, Large white gilts at estrus; MD, Mi gilts at diestrus; ME, Mi gilts at estrus. RPKM values are used to calculate the gene expression in RNA-seq and normalize the expression of one group to “1”. In real-time PCR, relative expression levels are calculated using ∆∆Ct value method and normalized by reference gene GAPDH, and similarly normalize the expression of one group to “1”. The data showed in Y axis represented the fold change. The unpaired Student’s t-test is used to evaluate the statistical signifcance of diﬀerences between the two groups, *P ≤ 0.05, **P ≤ 0.01. All data are presented as mean ± standard deviation (SD).
